# Characteristic Evaluation of Chameleon Luminophore Dispersed in Polymer

**DOI:** 10.3390/s20092623

**Published:** 2020-05-04

**Authors:** Miku Kasai, Yosuke Sugioka, Masanori Yamamoto, Takayuki Nagata, Taku Nonomura, Keisuke Asai, Yasuchika Hasegawa

**Affiliations:** 1 Department of Aerospace Engineering, Graduate School of Engineering, Tohoku University, 6-6-01 Aramakiaza-Aoba, Aoba-ku, Sendai, Miyagi 980-8579, Japan; sugioka.yosuke@aero.mech.tohoku.ac.jp (Y.S.); nagata@aero.mech.tohoku.ac.jp (T.N.); nonomura@aero.mech.tohoku.ac.jp (T.N.); asai@aero.mech.tohoku.ac.jp (K.A.); 2 Graduate School of Chemical Sciences and Engineering, Hokkaido University, North 13 West 8, Kita-ku, Sapporo Hokkaido 060-8628, Japan; m.yamamoto@cse.hokudai.ac.jp; 3 Institute for Chemical Reaction Design and Discovery (WPI-ICReDD), Hokkaido University, Sapporo, Hokkaido 001-0021, Japan; hasegaway@eng.hokudai.ac.jp; 4 Faculty of Engineering, Hokkaido University, Kita-13, Nishi-8, Sapporo, Hokkaido 060-8628, Japan

**Keywords:** temperature-sensitive paint (TSP), chameleon luminophore, sensitivity characteristics, polymer

## Abstract

A temperature-sensitive paint (TSP) using a chameleon luminophore ${\left[{\mathrm{Tb}}_{0.99}{\mathrm{Eu}}_{0.01}{\left(\mathrm{hfa}\right)}_{3}\left(\mathrm{dpbp}\right)\right]}_{n}$ is proposed. The chameleon luminophore was dispersed in isobutyl methacrylate polymer in a toluene solvent to fix it on a sample coupon. Temperature and pressure sensitivities of the chameleon luminophore-based TSP were measured using a spectrofluorophotometer. The emission for each wavelength was confirmed to be dependent on the temperature and pressure. The temperature and pressure sensitivities of the TSP were 0.81–2.8%/K and 0.08–0.12%/kPa, respectively. Higher temperature sensitivity can be obtained using the ratio of emissions from the two lanthanide ions, ${\mathrm{Tb}}^{\mathrm{III}}$ and ${\mathrm{Eu}}^{\mathrm{III}}$. The temperature sensitivity when using the ratio of the emission intensities at 616 nm derived from ${\mathrm{Eu}}^{\mathrm{III}}$ and at 545 nm derived from ${\mathrm{Tb}}^{\mathrm{III}}$ was 3.2%/K, which was the highest value in the present study. In addition, the pressure sensitivity for the case using the ratio of the emission intensities at 616 and 545 nm was $4.8\times 10^{-2}\%$/kPa. Higher temperature sensitivity and lower pressure sensitivity than that with a single wavelength can be achieved using the ratio of the emission intensities at the two peak wavelengths derived from ${\mathrm{Tb}}^{\mathrm{III}}$ and ${\mathrm{Eu}}^{\mathrm{III}}$.

## 1. Introduction

Temperature is one of the fundamental parameters that widely govern physical phenomena, chemical reactions, and life activities. Temperature measurement is used in a wide range of applications for the classification and control of phenomena. In the fields of engineering, temperature measurement is important because temperature distributions are deeply related parameters for friction drag, heat flux, and aerodynamic heating [1,2,3].

Thermocouples [4] and resistance thermometers have been conventionally used for the temperature measurement of models in wind tunnel tests. These are all point sensors that can only measure the temperature at the points where the sensors are located. It is difficult to measure the local temperature changes with the point sensors. Furthermore, installing several thermocouples and resistance thermometers requires both cost and space.

There are several ways to acquire the surface temperature distribution on an object. Infrared cameras [5] and temperature-sensitive liquid crystals [6,7,8,9] have typically been used for surface temperature measurements in wind tunnel tests. However, it is difficult for infrared cameras to measure transparent objects with the low infrared transmission, such as acrylics and water. Infrared cameras also have other problems such as being affected by radiation and reflections from other than those to be measured. Temperature-sensitive liquid crystals have problems such as that the temperature measurement range is narrow, and the color development of the liquid crystal changes depending on the illumination angle.

Temperature-sensitive paint (TSP) has been studied as an alternative measurement technique to solve these problems. Temperature-sensitive paint is generally composed of a polymer-based binder and a dye, which is excited at a specific wavelength of light and emits luminescence according to the temperature [10,11]. The surface temperature distribution with high spatial resolution can be obtained by measuring the emission of TSP by photoelectric sensors, such as a charge-coupled device (CCD) camera. TSP can be used even in extreme conditions. Heat flux distributions have been measured with TSP in short-duration hypersonic shock tunnels [1,2,3,12,13,14]. By estimating the heat flux based on the TSP result, the detection of laminar-turbulent boundary layer transition is also possible in cryogenic conditions [15,16,17].

A conventional TSP measurement requires wind-off reference images for correction of the nonuniformity of the paint to get quantitative temperature evaluation. When an object moves during a wind tunnel test, it is impractical to take a reference image for each position of the object during measurement. A lifetime method and bi-luminophore TSP can measure the temperature on a moving object without reference images. The temperature distributions on helicopter rotor blades and turbocharger compressor blades were successfully measured by TSP using a single-shot lifetime method [18,19]. Bi-luminophore TSP, which is a mixture of temperature-independent dyes and temperature-dependent dyes, can measure the temperature distribution on a moving object in principle [20,21]. However, the emission intensities of bi-luminophore paint decrease due to the photochemical interference between the two dyes. In addition, when the excitation wavelength of one dye and the emission wavelength of the other are the same in bi-luminophore paint, the nonuniformity of the photodegradation is caused.

Miyata et al. [22] developed a chameleon luminophore, which consists of polymer molecules that contain two lanthanide ions, europium ${\mathrm{Eu}}^{\mathrm{III}}$, which emits red light, and terbium ${\mathrm{Tb}}^{\mathrm{III}}$, which emits green light. The energy transfer between the two lanthanide ions is related to the activation energy via the triplet state of the linker molecule. Therefore, the energy transfer between the two lanthanide ions is controlled and temperature-dependent. At low temperature, the emission of the chameleon luminophore is green as the emission of ${\mathrm{Tb}}^{\mathrm{III}}$ is dominant. The emission color of the chameleon luminophore changes from yellow to red as the temperature increases as the emission of ${\mathrm{Eu}}^{\mathrm{III}}$ becomes dominant. The temperature of the object to which TSP is applied can be estimated from the ratio of the green and red emission intensities.

The chameleon luminophore has two distinctive features from bi-luminophore TSP: the first is that two lanthanide ions are coordinated in a single polymer molecule, and the second is that the energy transfer between the two ions is related to the activation energy [23]. Due to these properties, the chameleon luminophore has the following three advantages. First, higher temperature sensitivity can be realized than conventional bi-luminophore TSP. Second, the nonuniformity of the two different emissions can be eliminated. Third, the photochemical interference of the photodegradation is not caused. Therefore, temperature measurements with a chameleon luminophore are not influenced by the nonuniformity of the excitation light source or the nonuniformity of paint, and thus, no wind-off reference image is required. The chameleon luminophore is considered to be able to measure the temperature of a moving object quantitatively.

In the previous study, the temperature characteristics of the chameleon luminophore were evaluated in the solid-state [22] because of its low solubility in organic solvents. It is necessary to dissolve and disperse the chameleon luminophore in a binder and to paint it on a model for application of the chameleon luminophore-based TSP to wind tunnel tests. In addition, the pressure sensitivity of the chameleon luminophore has not been clarified. The dyes of conventional TSP are sensitive to changes in oxygen concentration, which can lead to temperature measurement errors. In this study, a chameleon luminophore-based TSP was produced by dispersing the chameleon luminophore in a polymer, which had a high oxygen permeability. The pressure sensitivity of the chameleon luminophore-based TSP was investigated, as well as the temperature sensitivity using a spectrofluorophotometer. The feasibility of the chameleon luminophore-based TSP is discussed.

## 2. Materials and Methods

### 2.1. The Probe and Its Emission Mechanism

**Figure 1:**
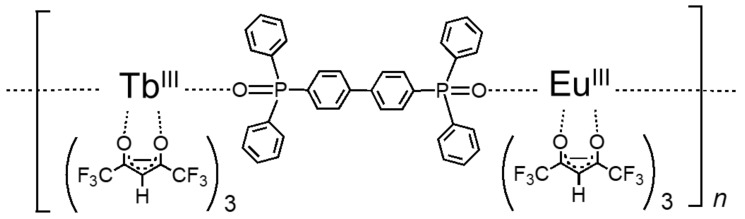
Chemical structure of the chameleon luminophore [22].

**Figure 2:**
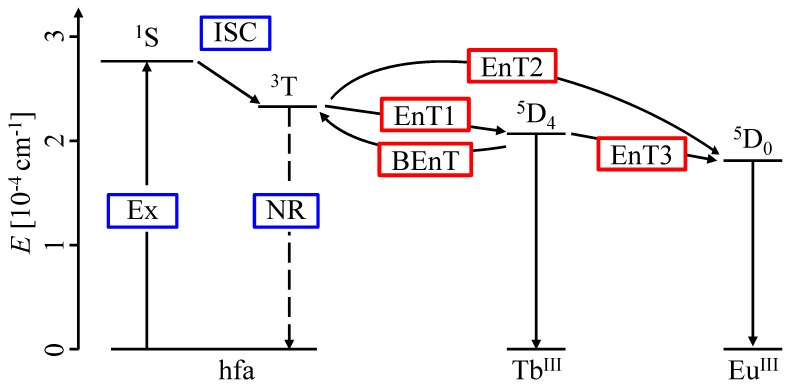
Energy transfer diagram of the chameleon luminophore (Ex: excitation, ISC: intersystem crossing, NR: nonradiative process, EnT: energy transfer, BEnT: back energy transfer) [24].

Figure 1 and Figure 2 show the chemical structure [22] and the energy transfer process [24] of the chameleon luminophore ${\left[{\mathrm{Tb}}_{0.99}{\mathrm{Eu}}_{0.01}{\left(\mathrm{hfa}\right)}_{3}\left(\mathrm{dpbp}\right)\right]}_{n}$ (hfa: hexafluoroacetylacetone, dpbp: 4,4’-bis(biphenylphosphoryl)biphenyl), respectively. This chameleon luminophore contained ${\mathrm{Tb}}^{\mathrm{III}}$ and ${\mathrm{Eu}}^{\mathrm{III}}$, which emitted green and red light, respectively, hfa ligands, and dpbp linker molecules. The molecular structure of the chameleon luminophore was thermally stable because it was a lanthanide coordination polymer. The lanthanide ion ${\mathrm{Tb}}^{\mathrm{III}}$ and the ligand hfa were chosen because the emission level of ${\mathrm{Tb}}^{\mathrm{III}}$ and the energy level of the triplet state of hfa were quite close. Moreover, the reason for incorporating ${\mathrm{Eu}}^{\mathrm{III}}$ was to make temperature measurement possible by taking the ratio of the emission intensities with ${\mathrm{Tb}}^{\mathrm{III}}$. Furthermore, since the energy transfer from ${\mathrm{Tb}}^{\mathrm{III}}$ to ${\mathrm{Eu}}^{\mathrm{III}}$ was temperature-dependent, a high temperature sensitivity could be achieved by measuring the temperature using the ratio of the emission intensities [22].

When the chameleon luminophore was excited, ${\mathrm{Tb}}^{\mathrm{III}}$ and ${\mathrm{Eu}}^{\mathrm{III}}$ were emitted by the following processes.

First, when hfa was excited to the triplet state (${}^{3}T$) and energy was transferred to the emission level of ${\mathrm{Tb}}^{\mathrm{III}}$ (EnT1) and ${\mathrm{Eu}}^{\mathrm{III}}$ (EnT2), then ${\mathrm{Tb}}^{\mathrm{III}}$ and ${\mathrm{Eu}}^{\mathrm{III}}$ emitted light. The triplet state of hfa was close to the emission level of ${\mathrm{Tb}}^{\mathrm{III}}$. Thus, the chameleon luminophore showed effective energy back transfer (BEnT) from the emission level of ${\mathrm{Tb}}^{\mathrm{III}}$ to the excited triplet state of hfa, which was a feature of the chameleon luminophore. BEnT depended on the energy barrier of the process; therefore, the emission intensity of ${\mathrm{Tb}}^{\mathrm{III}}$ ions with hfa ligands changed with temperature. The chameleon luminophore had high temperature sensitivity due to effective BEnT and EnT1 processes.

Second, the energy was transferred from the emission level of ${\mathrm{Tb}}^{\mathrm{III}}$ to that of ${\mathrm{Eu}}^{\mathrm{III}}$ (EnT3). This EnT3 energy transfer may also depend on the temperature. It was expected that the temperature could be measured quantitatively using the ratio of the emission intensities of the two lanthanide ions of the chameleon luminophore.

### 2.2. Sensor Fabrication

The chameleon luminophore, which appeared as a white powder, was synthesized using a previously reported procedure [22]. The chameleon luminophore was dispersible, but not soluble in organic solvents. The chameleon luminophore was particularly well dispersed in toluene so that toluene was used to mix it with the polymer.

Sample coupons were fabricated, and the temperature and pressure sensitivities of the chameleon luminophore in the polymer coating were evaluated using these coupons. Isobutyl methacrylate polymer, which had high oxygen permeability, was used as a binder to examine the pressure sensitivity of the chameleon luminophore. A coating material was prepared by mixing the chameleon luminophore, isobutyl methacrylate polymer, and toluene at a ratio of 90 mg:250 mg:20 mL. The mixture was stirred using an ultrasonic cleaner and allowed to stand in the dark for a day. The paint was stirred again using the ultrasonic cleaner and applied onto a 1 mm thick aluminum plate (50 × 30 mm) with a spray gun.

### 2.3. Experimental Setup

**Figure 3:**
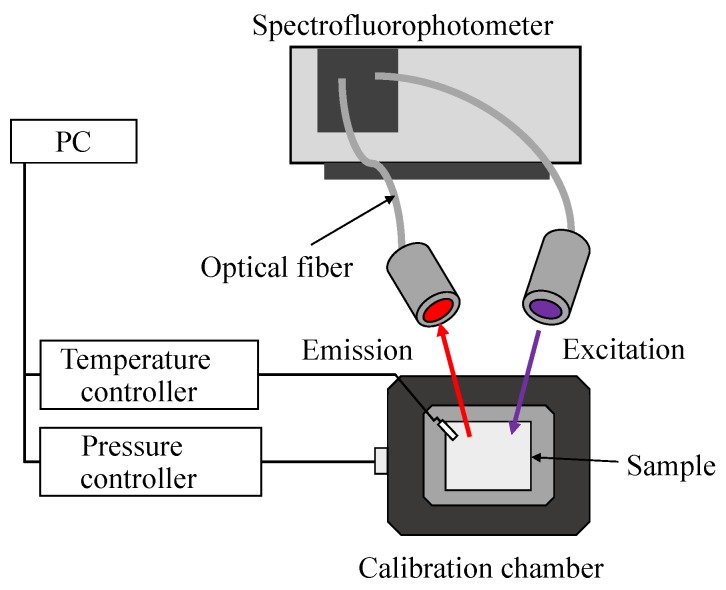
Schematic diagram of the measurement setup.

Figure 3 illustrates the experimental setup. A spectrofluorophotometer (RF-5300PC, Shimadzu Corporation (Kyoto, Japan)) was used to measure the excitation and emission of the sample coupons. The excitation light was guided from the excitation light irradiation source to the sample in the chamber by a light guide, and the emission of the sample coupons was guided from the chamber to the emission detector. A long-pass filter (440 nm, Y44, Hoya Candeo Optronics) was mounted in front of the emission detector. The signal-to-noise-ratio of the spectrofluorophotometer was 150 or more. This signal-to-noise-ratio was defined to be the ratio of the signal to the noise, whereas the signal and the noise were the emission intensity and the maximum variation of it at a Raman spectrum measured in distilled water, respectively. In this study, the measurement uncertainty caused by the noise was calculated with the signal-to-noise-ratio of 150. An error bar was drawn by calculating an error propagation equation.

Most application of temperature measurements was at the near-room temperature. The temperature characteristics of the chameleon luminophore-based TSP at the near-room temperature were investigated. The temperature *T* of the sample coupon was varied in the range of 273–333 K with a temperature controller (MT886-D1000, Thermoelectronics). The characteristics in this temperature range could be applied even in extreme conditions such as hypersonic wind tunnel tests because the temperature increment of a model surface was a few tens of degrees due to a few millisecond long test time [1,2,13,14]. The pressure *P* in the chamber was varied in the range of 5–140 kPa with a pressure controller (DPI515, Druck).

Emission spectra for each condition were measured once. In this measurement, it took approximately 0.25 s, and the emission intensity at each 1 nm wavelength was measured. The emission intensity at each 1 nm wavelength was the integrated value of the emission measured in this time. The emission spectra at the reference condition (P=100 kPa, T=293 K) was obtained repeatedly on the same sample coupon. The standard deviation of the ratio of the emission intensities was found almost equal to the measurement uncertainty calculated from the signal-to-noise-ratio of the spectrofluorophotometer. In addition, the reproducibility of the temperature and pressure sensitivities of the chameleon luminophore-based TSP was confirmed by comparing the data with those obtained with another sample in the ranges of 5–140 kPa at 293 K and 273–333 K at 100 kPa.

**Figure 4:**
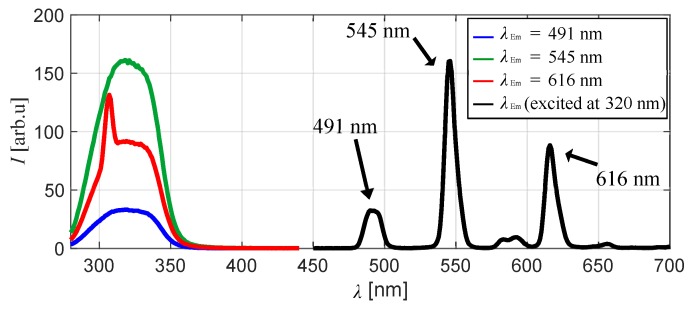
Excitation and emission spectra of the chameleon luminophore in the polymer.

Emission peaks of the chameleon luminophore in the polymer coating under reference conditions of $T_{\mathrm{ref}}=293$ K and $P_{\mathrm{ref}}=100$ kPa were detected at 491, 545, 583, 593, and 616 nm. The emission bands at 491, 545, and 616 nm were narrow. The emissions at 491 and 545 nm were derived from ${\mathrm{Tb}}^{\mathrm{III}}$, and the emission at 616 nm was derived from ${\mathrm{Eu}}^{\mathrm{III}}$. The emissions at 583 and 593 nm included both emissions from ${\mathrm{Tb}}^{\mathrm{III}}$ and ${\mathrm{Eu}}^{\mathrm{III}}$. The pressure and temperature sensitivities at the three wavelengths of 491, 545, and 616 nm, which were high emission intensity under the reference conditions, were evaluated. Figure 4 shows excitation spectra at these three peak wavelengths and an emission spectrum of the chameleon luminophore-based TSP excited at 320 nm. The excitation spectrum showed the excitation wavelength at which the emission of each wavelength was obtained. The excitation spectrum at an emission wavelength of 616 nm had a peak at 308 nm because of a peak subtransmission band. Excitation spectra at all three peak emission wavelengths, excluding the subtransmission band, had a peak at approximately 320 nm. Therefore, the excitation wavelength for this experiment was set to be 320 nm. Table 1 presents the settings used for the spectrofluorophotometer.

### 2.4. Analysis Method

The temperature and pressure sensitivity were calculated based on the emission intensity spectrum. The emission intensity I(λ) denoted the integrated value of the emission intensity *I* with a bandwidth of ±5 nm around the peak wavelength λ (nm). The emission intensity I(λ) was calculated under various conditions and was normalized with respect to the intensity under the reference condition $I{\left(\lambda \right)}_{\mathrm{ref}}$ at $T_{\mathrm{ref}}=293$ K and $P_{\mathrm{ref}}=100$ kPa. Here, the peak wavelengths were 491, 545, and 616 nm. The spectra in the reference condition (P=100 kPa, T=293 K) of every 15 cases were measured. The comparison of emission spectra at reference conditions showed that the effect of photodegradation was negligible. The relationship between the normalized emission intensity and temperature is empirically expressed by Equation (1). The relationship between the normalized emission intensity and normalized pressure $P/P_{\mathrm{ref}}$ is given by the Stern–Volmer equation (Equation (2)) [11].

(1) $$\frac{I(\lambda )}{I{\left(\lambda \right)}_{\mathrm{ref}}}=a_{1}T^{2}+b_{1}T+c_{1},$$

(2) $$\frac{I{\left(\lambda \right)}_{\mathrm{ref}}}{I(\lambda )}=A_{1}+B_{1}\frac{P}{P_{\mathrm{ref}}}.$$

The temperature and pressure sensitivities $S_{T1}(\%$/K) and $S_{P1}(\%$/kPa) were calculated by differentiating Equations (1) and (2) with respect to *T* and *P*, respectively. The present paper showed the temperature sensitivity $S_{T1}(\%$/K) and pressure sensitivity $S_{P1}(\%$/kPa) at $T_{\mathrm{ref}}=293$ K and $P_{\mathrm{ref}}$ = 100 kPa.

(3) $$S_{T1}={\left|\frac{d\left\{I\left(\lambda \right)/I{\left(\lambda \right)}_{\mathrm{ref}}\right\}}{dT}\right|}_{T=T_{\mathrm{ref}}}\times 100,$$

(4) $$S_{P1}=\left|B_{1}\right|.$$

Furthermore, the ratio of the emission intensities at the two peak wavelengths $I\left(\lambda_{1}\right)/I\left(\lambda_{2}\right)$ was calculated at each temperature and pressure, and these were normalized with respect to the ratio of the emission intensities at $T_{\mathrm{ref}}$ and $P_{\mathrm{ref}}$. The temperature and pressure changes were approximated by Equations (5) and (6), respectively.

(5) $$\frac{I\left(\lambda_{1}\right)/I\left(\lambda_{2}\right)}{{\left\{I\left(\lambda_{1}\right)/I\left(\lambda_{2}\right)\right\}}_{\mathrm{ref}}}=a_{2}T^{2}+b_{2}T+c_{2},$$

(6) $$\frac{{\left\{I\left(\lambda_{1}\right)/I\left(\lambda_{2}\right)\right\}}_{\mathrm{ref}}}{I\left(\lambda_{1}\right)/I\left(\lambda_{2}\right)}=A_{2}+B_{2}\frac{P}{P_{\mathrm{ref}}}.$$

Similar to Equations (3) and (4), the derivatives of Equations (5) and (6) with respect to *T* and *P* were the temperature sensitivity $S_{T2}(\%$/K) and pressure sensitivity $S_{P2}(\%$/kPa), respectively.

(7) $$S_{T2}={\left|\frac{d\left(\left\{I\left(\lambda_{1}\right)/I\left(\lambda_{2}\right)\right\}/{\left\{I\left(\lambda_{1}\right)/I\left(\lambda_{2}\right)\right\}}_{\mathrm{ref}}\right)}{dT}\right|}_{T=T_{\mathrm{ref}}}\times 100,$$

(8) $$S_{P2}=\left|B_{2}\right|.$$

## 3. Results and Discussion

### 3.1. Temperature Characteristics

**Figure 5:**
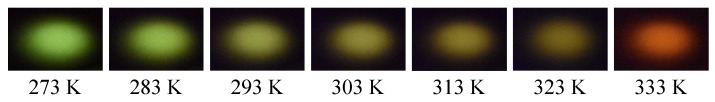
Photographs of the excited chameleon luminophore in the polymer at different temperatures taken by a single lens reflex camera (D3400, Nikon). The exposure time was 0.01 s. ISO sensitivity was 25,600. The aperture was f/6.3.

Figure 5 shows photographs of the excited chameleon luminophore in the polymer at different temperatures. The emission of the sample coupon was a green color at low temperature, which changed into yellow and red with an increase in the temperature, similar to the solid-state chameleon luminophore.

**Figure 6:**
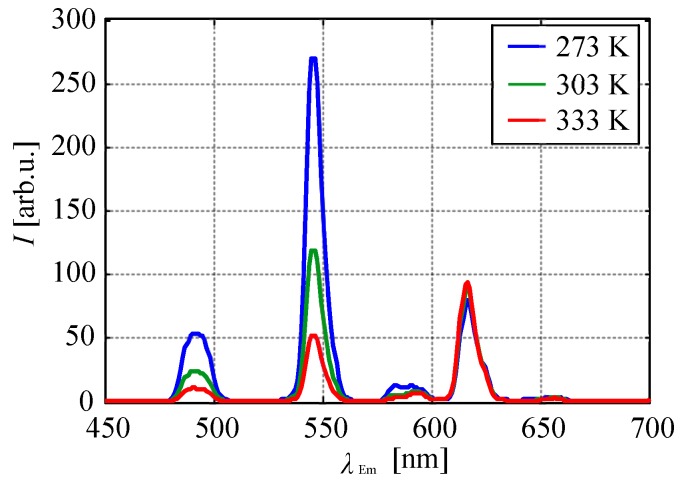
Effect of temperature on the emission spectrum of the chameleon luminophore in the polymer.

Figure 6 shows the change in the emission spectrum of the chameleon luminophore in the polymer coating at different temperatures. As the temperature increased, the emission intensity of ${\mathrm{Tb}}^{\mathrm{III}}$-derived emission peaks at 491 nm and that at 545 nm decreased, while the emission intensity of ${\mathrm{Eu}}^{\mathrm{III}}$-derived emission at 616 nm increased with the temperature. The emission intensities at 583 and 593 nm decreased with an increase in the temperature.

The energy transition diagram of the chameleon luminophore in Figure 2 shows the mechanism by which the emission intensity of ${\mathrm{Eu}}^{\mathrm{III}}$ increased with the temperature. The excitation energy of the chameleon luminophore transferred from the excited triplet hfa ligand to ${\mathrm{Tb}}^{\mathrm{III}}$ and ${\mathrm{Eu}}^{\mathrm{III}}$, and luminescence was emitted. The energy transitioned from ${\mathrm{Tb}}^{\mathrm{III}}$ to ${\mathrm{Eu}}^{\mathrm{III}}$, and this transition rate increased with the temperature [22,24]. Therefore, the emission intensity of ${\mathrm{Eu}}^{\mathrm{III}}$ increased with the temperature.

**Figure 7:**
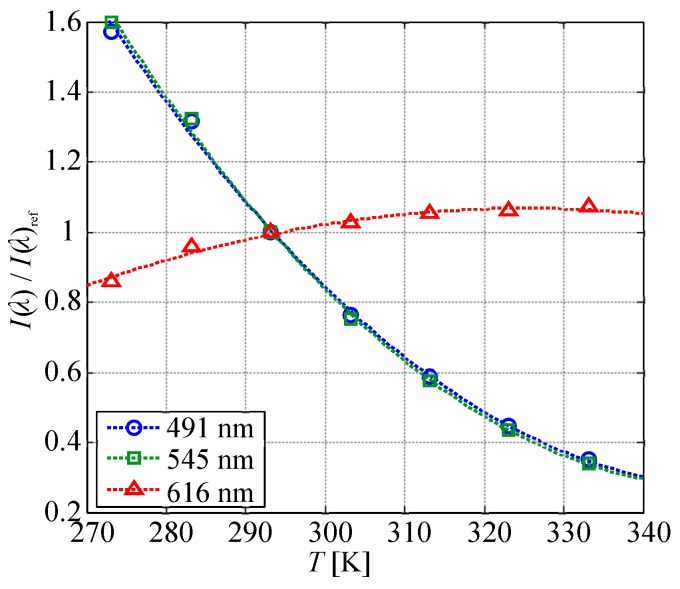
$I\left(\lambda \right)/I{\left(\lambda \right)}_{\mathrm{ref}}$ plotted as a function of temperature at P=100 kPa.

Figure 7 plots the relationship between the temperature and $I\left(\lambda \right)/I{\left(\lambda \right)}_{\mathrm{ref}}$. The measurement uncertainty of $I\left(\lambda \right)/I{\left(\lambda \right)}_{\mathrm{ref}}$ was approximately 0.7%, which was much smaller than the symbol size in the plot. The emission intensity at the peak wavelengths of 491 and 545 nm derived from ${\mathrm{Tb}}^{\mathrm{III}}$ showed almost the same decay rate as a function of the temperature. The approximate curve derived from Equation (1) is plotted as a dashed line. The temperature sensitivity at 293 K was calculated from the quadratic approximation curve, as shown in Figure 7. The emission intensities at the peak wavelengths of 491 and 545 nm derived from ${\mathrm{Tb}}^{\mathrm{III}}$ had the highest temperature sensitivity of 2.8%/K, and that at 616 nm derived from ${\mathrm{Eu}}^{\mathrm{III}}$ was 0.81%/K. On the other hand, some conventional TSP had temperature sensitivities of approximately 2.8–4.2%/K [25,26]. The temperature sensitivity of the chameleon luminophore at a single wavelength dispersed in the polymer was equivalent to that of conventional TSP.

**Figure 8:**
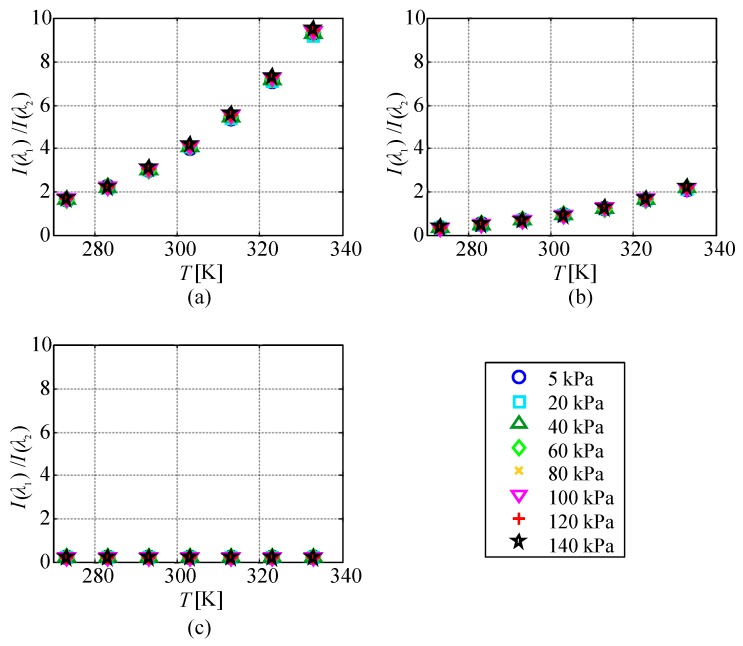
Temperature evolution of the ratio of the emission intensities of two wavelengths at each pressure. (**a**) $\lambda_{1}=616$ nm, $\lambda_{2}=491$ nm; (**b**) $\lambda_{1}=616$ nm, $\lambda_{2}=545$ nm; (**c**) $\lambda_{1}=491$ nm, $\lambda_{2}$ = 545 nm.

Figure 8 shows the relationship between the temperature and the ratio of the emission intensities at two wavelengths $I\left(\lambda_{1}\right)/I\left(\lambda_{2}\right)$ for each pressure condition. The measurement uncertainty in $I\left(\lambda_{1}\right)/I\left(\lambda_{2}\right)$ was at most 1%, which was much smaller than the symbol size. The ratio of the emission intensities at 616 and 491 nm and that at 616 and 545 nm increased with the temperature. However, the ratio of the emission intensities at 491 and 545 nm did not change significantly, even when the temperature increased.

When calculating the temperature from the ratio of the emission intensities at two peak wavelengths, the emission intensity of the two wavelengths should be at a similar signal-to-noise-ratio level. The ratio of the emission intensities at $\lambda_{1}=616$ nm and $\lambda_{2}=491$ nm was between 1.5 and 9.6 in the range of 273–333 K, and the emission intensity at 491 nm was smaller than that at 616 nm. Therefore, the shot noise detected by observation of the emission at 491 nm was dominant in the temperature measurement by the ratio of the emission intensities at the two wavelengths. On the other hand, the ratio of the emission intensities at $\lambda_{1}=616$ nm and $\lambda_{2}=545$ nm was between 0.3 and 2.3 in the range of 273–333 K. The ratio of the emission intensities at $\lambda_{1}=616$ nm and $\lambda_{2}=545$ nm showed the highest performance of all combinations in terms of shot noise.

**Figure 9:**
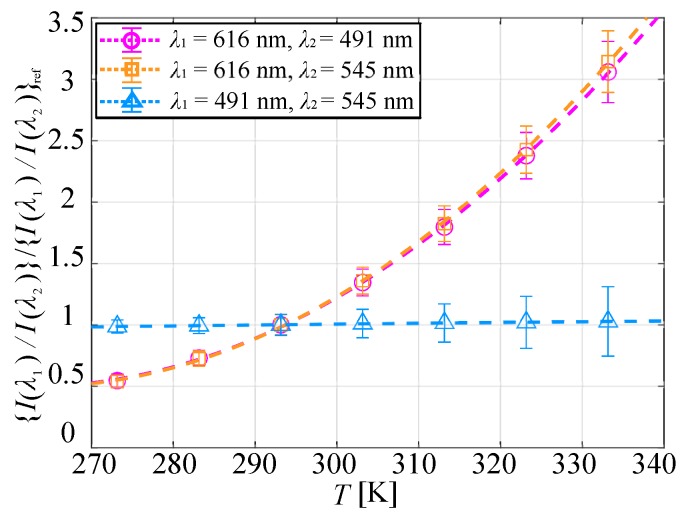
Normalized ratio of the emission intensities at two wavelengths plotted as a function of the temperature at P=100 kPa.

The ratio of the emission intensities at the two wavelengths took a wide range of values, making them unsuitable for direct comparison of temperature sensitivity. Therefore, it was necessary to normalize the ratio of the emission intensities, and the temperature sensitivities of the ratio of the emission intensities were compared. Figure 9 shows the relationship between the temperature and the normalized ratio of the emission intensities at the two wavelengths. Dashed lines in Figure 9 are the approximate curves derived from Equation (5), and the temperature sensitivity at 293 K was calculated from the quadratic approximation curve. The normalized ratio of the emission intensities at 616 and 491 nm and that of 616 and 545 nm were almost identical, with temperature sensitivities of 3.1%/K and 3.2%/K, respectively. Higher temperature sensitivity could be achieved when the ratio of the emission intensities at two peak wavelengths was used compared with a single wavelength.

On the other hand, the normalized ratio of the emission intensities at 491 and 545 nm was almost unity at all temperature conditions, and the temperature sensitivity was $8.3\times 10^{-2}$%/K. The emission intensities at the peak wavelengths of 491 and 545 nm had the same luminescence behavior with respect to the temperature because both peak wavelengths were emitted by the same emission level of ${\mathrm{Tb}}^{\mathrm{III}}$. The emissions at 491 and 545 nm derived from ${\mathrm{Tb}}^{\mathrm{III}}$ came from the same emission level ${}^{5}D_{4}$. There were three governing emission processes from ${}^{5}D_{4}$. The first one was a 4F-4F emission process (emission at 491 and 545 nm). The second one was energy transfers such as a back energy transfer of the hfa ligand to ${}^{3}T$, an energy transfer to ${\mathrm{Eu}}^{\mathrm{III}}$, and the intersystem crossing (ISC) of hfa. The third one was a nonradioactive process from ${}^{5}D_{4}$. The temperature sensitivities of 491 and 545 nm were almost identical since most of the quenching mechanisms were the same.

**Figure 10:**
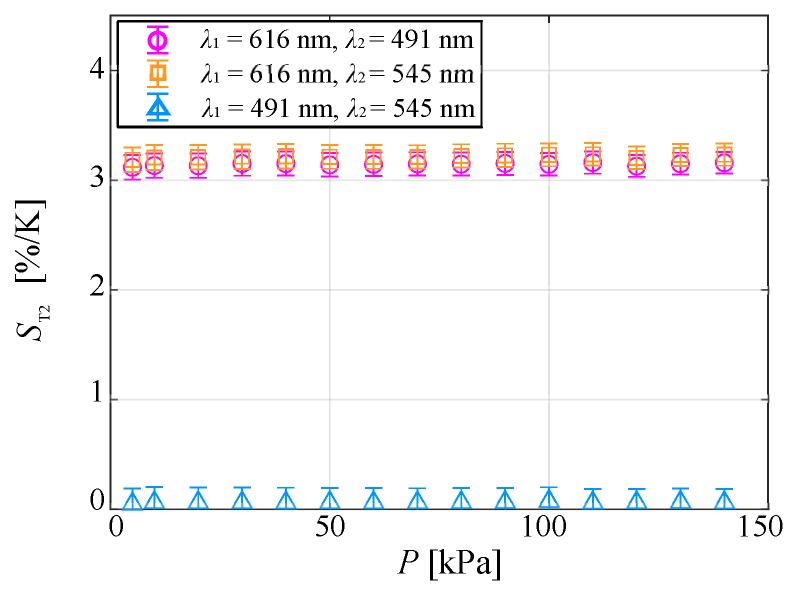
Temperature sensitivity plotted as a function of the ambient pressure.

Figure 10 shows the temperature sensitivity at each pressure condition. In all combinations of two wavelengths, $I\left(\lambda_{1}\right)/I\left(\lambda_{2}\right)$ had the same temperature sensitivity at any pressure. Therefore, the temperature could be measured from the ratio of the emission intensities at two peak wavelengths of the chameleon luminophore without being affected by the pressure in the range of 5–140 kPa.

The emission intensities at the peak wavelengths of 616 and 545 nm had the highest temperature sensitivity of all the combinations; therefore, this combination was most suitable for temperature measurements.

### 3.2. Pressure Characteristics

**Figure 11:**
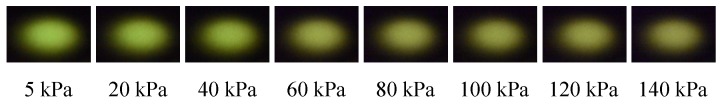
Photographs of the excited chameleon luminophore in the polymer at different pressures taken by a single lens reflex camera (D3400, Nikon). The exposure time was 0.01 s. ISO sensitivity was 25,600. The aperture was f/6.3.

Figure 11 shows the chameleon luminophore in the polymer binder, the color of which changed with the ambient pressure. The emission of the sample coupon was green colored at low pressure, which changed to yellow with an increase in the pressure.

**Figure 12:**
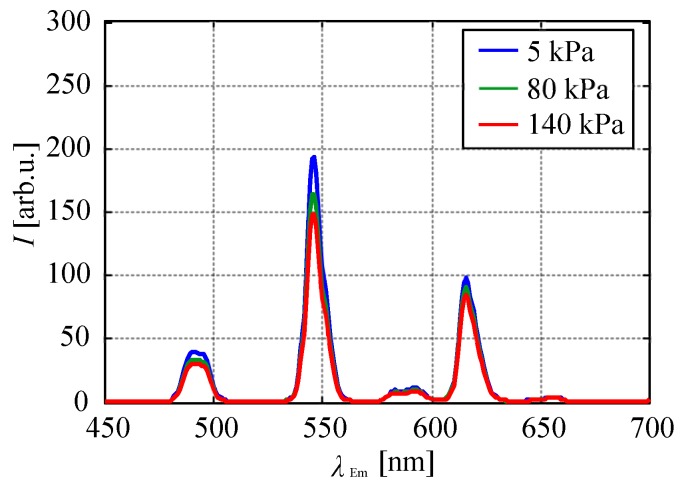
Effect of pressure on the emission spectrum of the chameleon luminophore in the polymer.

**Figure 13:**
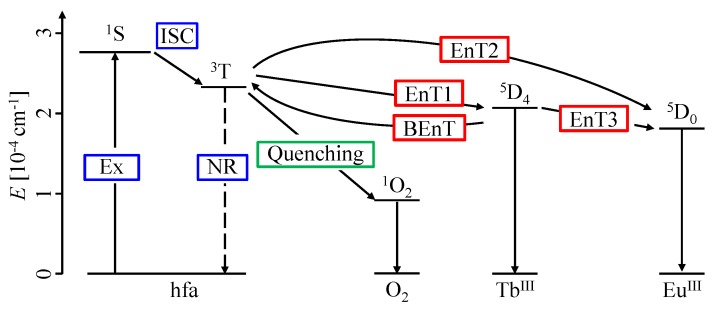
Energy transfer diagram of the chameleon luminophore when oxygen quenching occurs.

Figure 12 shows the change in the emission spectrum of the chameleon luminophore in the polymer coating at different ambient pressures. The emission intensity of all emission peaks decreased with an increase in the ambient pressure. As previously stated, this decrease in emission intensities was not caused by photodegradation. This study first revealed the dependence of the emission intensity on the ambient pressure for the chameleon luminophore in a polymer binder. We discuss the mechanism by which the emission intensity of ${\mathrm{Tb}}^{\mathrm{III}}$ and ${\mathrm{Eu}}^{\mathrm{III}}$ decreased with an increase in the ambient pressure from the energy transition diagram of the chameleon luminophore shown in Figure 13. The hfa ligand was quenched with oxygen when hfa was excited and in the triplet state (${}^{3}T$), and thus, the amount of energy transitioned from hfa to ${\mathrm{Tb}}^{\mathrm{III}}$ and ${\mathrm{Eu}}^{\mathrm{III}}$ decreased. As a result, the emission intensities of all the emission wavelengths decreased.

**Figure 14:**
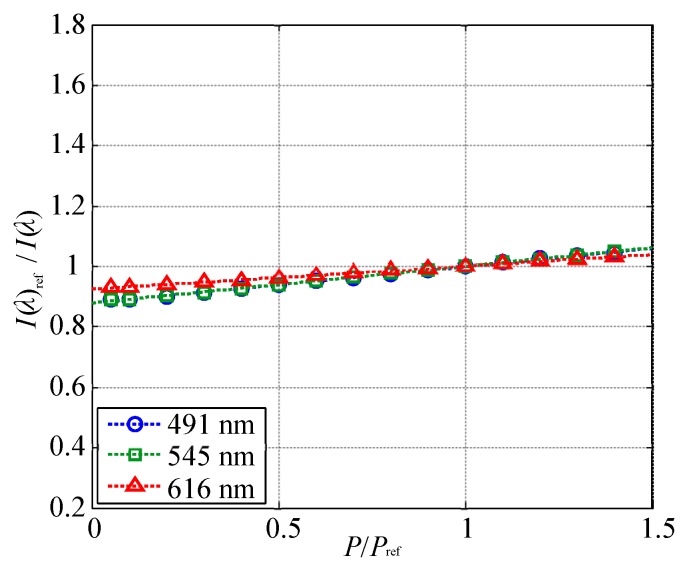
$I{\left(\lambda \right)}_{\mathrm{ref}}/I\left(\lambda \right)$ as a function of the normalized pressure at T=293 K.

Figure 14 presents the relationship between the normalized pressure and $I{\left(\lambda \right)}_{\mathrm{ref}}/I\left(\lambda \right)$. The measurement uncertainty of $I{\left(\lambda \right)}_{\mathrm{ref}}/I\left(\lambda \right)$ was approximately 0.7%, which as much smaller than the symbol size. This figure shows that the emission intensity at the peak wavelengths of 491 and 545 nm derived from ${\mathrm{Tb}}^{\mathrm{III}}$ showed almost the same decay rate as a function of the pressure, and the approximate curve derived from Equation (2) is plotted as a dashed line. The pressure sensitivity at 293 K was calculated by a linear approximation. The emission intensities at the peak wavelengths of 491 and 545 nm derived from ${\mathrm{Tb}}^{\mathrm{III}}$ had the highest pressure sensitivity of 0.12%/kPa, and that of 616 nm derived from ${\mathrm{Eu}}^{\mathrm{III}}$ was $7.7\times 10^{-2}$%/kPa.

The triplet state of hfa was closer to the emission level of ${\mathrm{Tb}}^{\mathrm{III}}$ and ${\mathrm{Eu}}^{\mathrm{III}}$ than the singlet state of oxygen (${}^{1}O_{2}$). Therefore, the energy was more easily transferred from hfa to ${\mathrm{Tb}}^{\mathrm{III}}$ and ${\mathrm{Eu}}^{\mathrm{III}}$ than from hfa to oxygen; the magnitude of the pressure sensitivity of the chameleon luminophore was less than that of its temperature sensitivity. On the other hand, the pressure sensitivities of conventional TSP were less than 0.01%/kPa, whereas the pressure sensitivity of the chameleon luminophore at the emission wavelength derived from ${\mathrm{Tb}}^{\mathrm{III}}$ was slightly higher than that of the conventional TSP.

**Figure 15:**
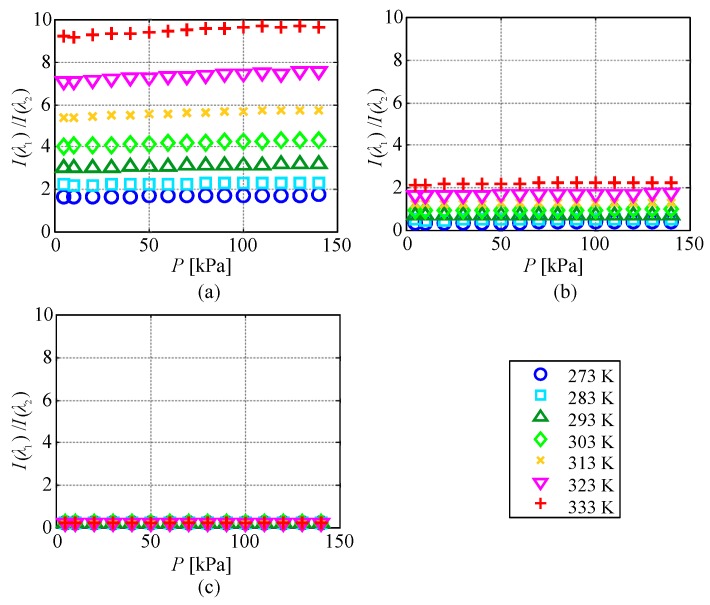
Ambient pressure evolution of the ratio of the emission intensities at two wavelengths for each temperature condition. (**a**) $\lambda_{1}=616$ nm, $\lambda_{2}=491$ nm; (**b**) $\lambda_{1}=616$ nm, $\lambda_{2}=545$ nm; (**c**) $\lambda_{1}=491$ nm, $\lambda_{2}=545$ nm.

Figure 15 presents the relationship between the pressure and the ratio of the emission intensities at two wavelengths $I\left(\lambda_{1}\right)/I\left(\lambda_{2}\right)$. The measurement uncertainty in $I\left(\lambda_{1}\right)/I\left(\lambda_{2}\right)$ was at most 1%, which was much smaller than the symbol size. The ratio of the emission intensities of all combinations did not change significantly, even when the ambient pressure rose. Therefore, there was no significant impact of the ambient pressure on $I\left(\lambda_{1}\right)/I\left(\lambda_{2}\right)$.

**Figure 16:**
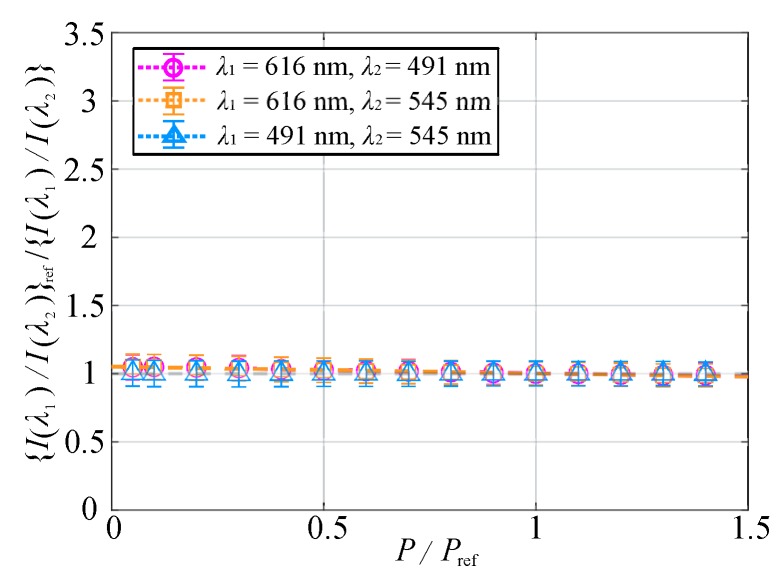
Normalized ratio of the emission intensities at two wavelengths plotted against the normalized pressure at T=293 K.

The ratio of the emission intensities was normalized, and the pressure sensitivities in the ratio of the emission intensities were compared for convenience. Figure 16 shows the relationship between the normalized pressure and the normalized ratio of the emission intensities at the two wavelengths. The pressure sensitivity was calculated based on a linear approximation curve. The normalized ratio of the emission intensities at 616 and 491 nm and that at 616 and 545 nm were almost identical with pressure sensitivities of $4.7\times 10^{-2}\%$/kPa and $4.8\times 10^{-2}\%$/kPa, respectively. On the other hand, the normalized ratio of the emission intensities at 491 and 545 nm was approximately unity regardless of the ambient pressure, and its pressure sensitivity was $1.3\times 10^{-3}\%$/kPa. When the ratio of the emission intensities at two peak wavelengths was used, a lower pressure sensitivity could be achieved than that at a single wavelength.

**Figure 17:**
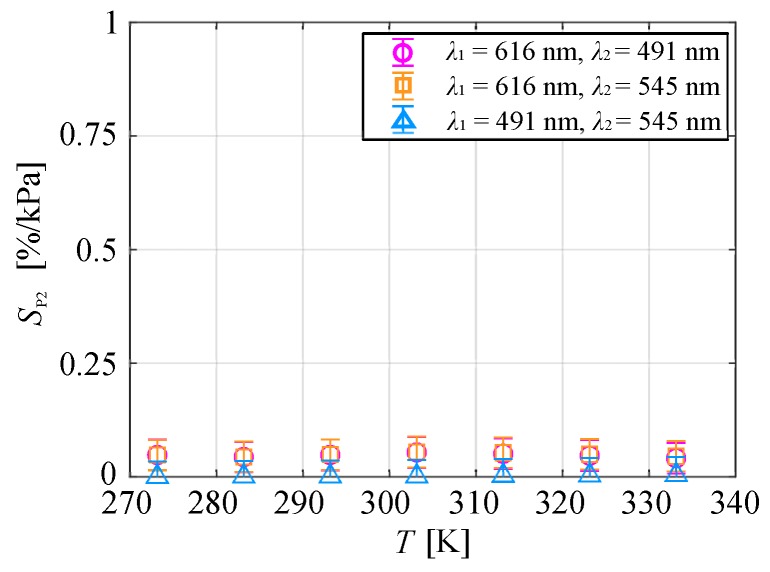
Pressure sensitivity as a function of the temperature.

Figure 17 shows the pressure sensitivity of the normalized ratio of the emission intensities at two peak wavelengths for each temperature condition. The pressure sensitivity hardly changed with temperature for any combination of wavelengths and was less than 0.1% in the measurement range of 273–333 K. The pressure sensitivity was small despite using isobutyl methacrylate polymer binders with high oxygen permeability, and the temperature dependence of pressure sensitivity was negligible. Therefore, the temperature could be measured from the ratio of the emission intensities at two peak wavelengths of the chameleon luminophore without being affected by the pressure in the range of 5–140 kPa.

Additionally, isobutyl methacrylate polymer, which had high oxygen permeability, was used as a binder, and the pressure sensitivity of the chameleon luminophore dispersed in a polymer was examined. The results suggested that TSP with lower pressure sensitivity could be realized by dispersing the chameleon luminophore in a polymer with lower oxygen permeability.

## 4. Conclusions

The chameleon luminophore consisted of two lanthanide ions coordinated in a single polymer molecule. The temperature on a moving object could be measured by taking the ratio of the emission of different wavelengths emitted from two lanthanide ions. The two ions were coordinated at the atomic level, which could eliminate the nonuniformity of the paint, which was a problem in conventional bi-luminophore TSP. The energy transfer between two ions in the chameleon luminophore was via the linker molecules and was temperature-dependent. Therefore, the problems of the nonuniformity of photodegradation and the emission intensity decrease due to photochemical interference between two dyes did not occur.

Although the temperature characteristics of the chameleon luminophore in the solid-state were studied, its pressure sensitivity has not been clarified. Furthermore, the chameleon luminophore has never been applied as TSP. In the present study, the first chameleon luminophore-based TSP was produced by dispersing in isobutyl methacrylate polymer binders with high oxygen permeability. The temperature and pressure characteristics of the chameleon luminophore-based TSP were investigated using a spectrofluorophotometer. The main emission wavelengths of the chameleon luminophore were 491 and 545 nm from ${\mathrm{Tb}}^{\mathrm{III}}$ and 616 nm from ${\mathrm{Eu}}^{\mathrm{III}}$.

This study showed that the temperature measurement using the chameleon luminophore-based TSP was practical in several points. The chameleon luminophore was a single molecule so that it could potentially solve the problems caused by photodegradation and the nonuniformity of the ratio of the emission intensities. In addition, each peak emission of the paint had a narrow emission band so that it did not overlap with other emissions, allowing us to use a color camera for measurement emissions. It is also worth mentioning that temperature could be distinguished at a glance from the color of the emitted light.

The chameleon luminophore dispersed in a polymer was clarified to have a pressure sensitivity. The temperature and pressure sensitivity of the emission peak wavelength derived from ${\mathrm{Tb}}^{\mathrm{III}}$ were 2.8%/K and 0.12%/kPa, respectively. The temperature sensitivity of the emission peak wavelength derived from ${\mathrm{Eu}}^{\mathrm{III}}$ was 0.81%/K, and the pressure sensitivity was $7.7\times 10^{-2}$%/kPa.

When the ratio of the emission intensities at two peak wavelengths was taken, the temperature sensitivity was higher and the pressure sensitivity lower than those with a single wavelength emission intensity. The temperature sensitivity of the ratio of the emission intensities at 616 and 545 nm was 3.2%/K, and the pressure sensitivity was $1.3\times 10^{-3}\%$/kPa. The results indicated that quantitative temperature measurement, even for moving objects in wind tunnel tests, could be achieved by taking the ratio of the emission intensities at two peak wavelengths, 616 and 545 nm. The chameleon luminophore-based TSP could potentially solve the problems of the conventional bi-luminophore TSP. Our next goal was to confirm the practicality of the proposed TSP, and we plan to apply it to aerodynamic tests as the next step.

## Figures and Tables

**Table 1:** Spectrofluorophotometer settings.

Excitation wavelength *$\lambda_{\mathrm{Ex}}$*(nm)	320
Fluorescence spectrum range *$\lambda_{\mathrm{Em}}$*(nm)	450–700
Slit width of excitation/fluorescence spectroscope (nm)	5
Sampling interval (nm)	1
